# The Impact of the SARS-CoV-19 Pandemic on the Global Gross Domestic Product

**DOI:** 10.3390/ijerph18105246

**Published:** 2021-05-14

**Authors:** Piotr Korneta, Katarzyna Rostek

**Affiliations:** Faculty of Management, Warsaw University of Technology, 02-524 Warszawa, Poland; katarzyna.rostek@pw.edu.pl

**Keywords:** SARS-CoV-19, pandemic, crisis management, GDP, gross domestic product, multi-segment Theil–Sen

## Abstract

The rapid, unexpected, and large-scale expansion of the SARS-CoV-19 pandemic has led to a global health and economy crisis. However, although the crisis itself is a worldwide phenomenon, there have been considerable differences between respective countries in terms of SARS-CoV-19 morbidities and fatalities as well as the GDP impact. The object of this paper was to study the influence of the SARS-CoV-19 pandemic on global gross domestic product. We analyzed data relating to 176 countries in the 11-month period from February 2020 to December 2020. We employed SARS-CoV-19 morbidity and fatality rates reported by different countries as proxies for the development of the pandemic. The analysis employed in our study was based on moving median and quartiles, Kendall tau-b coefficients, and multi-segment piecewise-linear approximation with Theil–Sen trend lines. In the study, we empirically confirmed and measured the negative impact of the SARS-CoV-19 pandemic on the respective national economies. The relationship between the pandemic and the economy is not uniform and depends on the extent of the pandemic’s development. The more intense the pandemic, the more adaptive the economies of specific countries become.

## 1. Introduction

The SARS-CoV-19 pandemic has rapidly spread around the world and has posed significant threats to public health, as well as social and economic standing of many countries. The unexpected onset of the global health crisis caused many scholars to change their research domains towards pandemic-related areas. A significant number of studies have already been conducted in terms of the medical aspects of the coronavirus-2019, including the understanding of coronavirus-2019 pathogenic mechanism [1], its transmission routes [2], and different patterns of symptom development [3,4,5,6]. Several researchers have already identified obesity; ageing; and comorbidities such as cardiovascular diseases, cancers, and diabetes as risk factors in SARS-CoV-19 patients [7,8,9,10]. The SARS-CoV-19 fatality rate is estimated at around 4% of infected patients but varies between respective countries from 0 to 20% [11,12]. Such a large range of fatality discrepancies might result from different demographic, economic, and political variables specific to different countries [12]. Finally, it is noteworthy that thanks to the extensive work performed by scientists and pharmaceutical companies, the vaccination process is already underway around the world [13].

As research in the healthcare perspective is starting to yield significant effects, it is now time to focus our attention on the economy and the significant consequences the pandemic has had in this context. A number of scholars have already studied the impact of the global health crisis on financial markets whose sharp fluctuations during the pandemic have led to confusion and uncertainty among investors [14,15,16]. Results reported by other researchers indicate that the SARS-CoV-19 pandemic has changed consumer behaviors [17]. The first papers on the impact of the SARS-CoV-19 pandemic on the condition of the healthcare industry have also already appeared. It has been claimed that the pandemic has had both positive and negative effects on respective branches of the healthcare industry, but the latter are nonetheless predominant [18]. The negative impact of the pandemic on the economies of various countries has already been acknowledged in both local [19,20,21] and global terms [22,23,24]. The first gross domestic product (GDP) forecasts have also been already provided [25]. It is believed that the decline of global economy stems from two primary factors [23]. The first relates to the various forms of isolation imposed by the governments, such as social distancing, shutdowns of events and corporate offices, and lockdowns. The second is the uncertainty about how bad the situation can get. According to information provided by the International Monetary Fund (IMF), the global gross domestic product is estimated to have declined by around −3.5%. We should note, however, that the data provided by IMF indicate significant discrepancies between economic effects observed in respective countries [26]. Such differences may result from two major factors. Firstly, the degree to which the economies of specific countries have been affected by the pandemic. This can be measured relative to SARS-CoV-19 morbidity and fatality rates. The second factor relates to the various restrictions introduced in particular countries [27,28], availability of public aid [29,30], and interconnections developed between the economies of various countries during the pandemic [31,32].

Since 2020 is already over and real data reflecting changes in the global GDP are now available, scientists can commence studies in this area. The objective of our paper was to consider the impact of the SARS-CoV-19 pandemic on national economies. We selected SARS-CoV-19 morbidity and fatality rates as viable proxies of pandemic development in specific countries, while GDP fluctuations served as a measure of the economy’s condition. In order to achieve the objective of the paper, we formulated the following two hypotheses:

**H1:** *There is a negative correlation between the SARS-CoV-19 pandemic development and GDP growth.*

**H2:** *The relationship between the pandemic and GDP fluctuations is not a linear one.*

The first of the formulated hypotheses is based on the postulates of other scholars [22,23,24] who reported on the negative effects of the pandemic on the global economy. The second results from the assumption that the actions taken by governments might affect the severity of the impact that the pandemic has had on the economies of respective countries [27,28,29,30,31,32]. To our best knowledge, this study is the first to explore correlations between SARS-CoV-19 morbidity and fatality rates on the one hand, and the economies of specific countries on the other. Notably, the objective of this paper was not to analyze the sources of morbidity and fatality rates discrepancies between respective countries, but rather to study how such differences affect their economies.

The paper analyzed data relating to 176 countries from six different continents in a 11-month period from February 2020 to December 2020. Because of significant data dispersion, we had to use robust statistical methods in our analysis. We employed moving medians and quartiles to determine the form of the relationship between the degree of pandemic development and the change of gross domestic product in specific countries, in their respective ranges. We employed the Kendall tau-b to determine the significance of correlations. Next, we calculated multi-segment Theil–Sen linear approximations to fit the obtained relations.

The rest of the paper is structured in the following way: In Section 2, research methods and their different components such as the database and sample of observations, variables, and design of the study are provided. Section 3 presents results derived from empirical data. Section 4 provides the discussion and indicates limitations of our study. Finally, we provide conclusions and implications.

## 2. Materials and Methods

### 2.1. Database and Sample of Observations

The data used in this study were obtained from two well recognized sources: the open access database “Our World in Data” [33] and the “International Monetary Fund” [26]. From the latter, information on the change of gross domestic product in 2020 as of 7 March 2021 as compared to 2019 by different countries was obtained. The “Our World in Data” database compiles official information relating to the SARS-CoV-19 pandemic, including the number of reported new SARS-CoV-19 cases and deaths and PCR testing around the world [33]. From this database, accessed on March 7, 2021, we obtained 59,787 daily observations relating to 176 countries in a 11-month period from February 2020 to December 2020. Since this database provided no information regarding other countries, we were not in a position to analyze all the countries worldwide. We aggregated the obtained daily observations into 176 yearly observations. As a result, the studied figures comprised 82.5 million and 1.8 million diagnosed SARS-CoV-19 cases and reported deaths, respectively, and related the same to the world population of 7.6 billion. Table 1 presents the breakdown of observation samples used in the study, divided by continent, with the numbers of SARS-COV-19 new cases and deaths reported and the respective number of countries considered.

### 2.2. Variables

The variables used in this study, their acronyms, number of observations, and descriptions of calculations are presented in Table 2. The first two of the studied variables are SARS-CoV-19 case rate (CCR) and SARS-CoV-19 fatality rate (CFR). We calculated these variables for each country. Since the populations of respective countries differ, we divided newly diagnosed SARS-CoV-19 cases and deaths per 1000 residents. A similar approach has already been used by other scholars [34]. We employed CCR and CFR variables to measure the pandemic. The last of the variables selected for the study was the change in gross domestic product relative increase (GDP). This ratio indicates the market value of all goods and services produced in a specific period. Hence, the change in GDP relative increase is a good proxy of the change in the world’s economy. 

### 2.3. Design and Data Analysis

Once the variables had been selected, we analyzed descriptive statistics. In the next step of the study, we plotted the relationships between respective variables on graphs. Visualization of the contemplated relationships was achieved by employing the moving median and first and third quartiles in order to determine different correlations between the considered variables in different ranges [35]. We calculated the median values for both variables in a moving window containing data from 50 observations. The division of our relations into segments helped us to identify ranges of pandemic intensity with different effects on the GDP. In practice, it is important to identify what pandemic intensity has a dominant effect on the change of global gross domestic product. The approach of dividing infected individuals according to the 80/20 rule has been suggested to explain transmission events during the SARS epidemic [36]. After identifying the relation between variables in different ranges, we calculated the Kendall tau-b [37,38,39] coefficients. Kendall tau-b rank correlation coefficient is widely used as a distribution-free measure of the strength and direction of association that exists between two variables. In order to analyze data using Kendall tau-b, one should measure two variables on an ordinal or continuous scale (interval or ratio variables), and data should follow a monotonic relationship. We calculated Kendall tau-b rank correlation coefficient in intervals where monotonic relationship was indicated by the moving median and quartiles. Our variables thus satisfy the requirements of Kendall tau-b, which can be used to determine the strength and direction of the association [40,41,42,43]. The values of Kendall’s rank correlation coefficient around 0.1 are typically obtained in the analysis of meteorological data, where they vary between 0.012 and 0.159 [44]. Decision about the statistical significance was made using empirical significance level (*p*-value) compared to α = 5%. We used one-tailed Mann–Kendall test [41,42].

Next, we applied the multi-segment linear regression with the Theil–Sen robust lines to characterize different relations between our independent and dependent variables in different ranges [45]. This regression model was selected because it is resistant to the effects of outliers and non-normality in residuals. The multi-segment model is the most suitable choice if more than one regression relation is necessary to characterize different relations that may occur in different regimes of explanatory variables. The nonparametric estimate of the slope of the line, identified as the Theil–Sen (or Kendall–Theil or Sen) slope in the literature, is calculated as the median of all possible pairwise slopes between points [45,46,47,48,49]. The line intercept was calculated by using the median slope and the median of the independent and dependent variables [47,49]. The multi-segment Theil–Sen regression technique was comprehensively described by Granato [50] and has been used by other researchers [50,51]. Furthermore, the Theil–Sen estimator is considerably more accurate than non-robust simple linear regression, especially for heteroskedastic and skewed data. It should also be noted that the Theil–Sen estimator provides better results than the non-robust least squares method, even for normally distributed data [47]. Finally, we obtained multi-segment piecewise-linear approximation to the studied relationships between considered variables. The Theil–Sen procedure has already been used in other studies in the fields of economy [51] and health [52,53].

## 3. Results

Presented in Table 3 are the descriptive statistics of the variables used in this study, calculated for each country in the relevant period. On average, 15.749 (CCR) per 1000 persons were infected with SARS-CoV-19 in 2020, and 76.819 was the maximum. Great differences in mortality between respective countries are revealed in the min, median, and max values of the SARS-CoV-19 fatality rate (CFR). The highest mortality of 1.739 deaths per 1000 people was reported in San Marino, closely followed by Belgium, where the fatality rate in the studied period reached 1.685 per 1000. The (arithmetic) mean of the GDP fluctuation was −5.694 (%) and was negative in 153 countries, with only 23 states reporting positive values.

Table 4 presents the results of statistical tests for the relationships between CCR, CFR, and GDP in two identified ranges for each variable. We showed the Kendall tau-b values with their *p*-values (one side). In the selected ranges, we performed linear approximation of the studied relationships using the Theil–Sen procedure. The obtained values of Theil–Sen slopes (m) and intercepts (b) are given in Table 4. This two-segment piecewise-linear approximation well describes the considered relationships in the two identified ranges.

**Figure 1:**
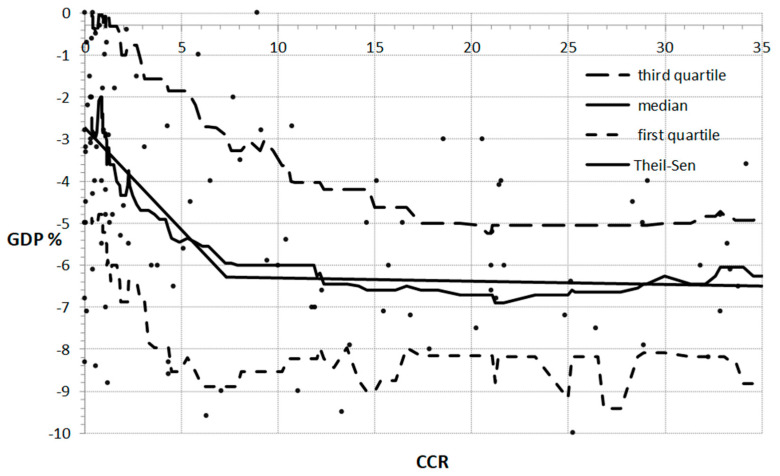
The relation between CCR and GDP with the moving median, first and third quartiles, and two-segment linear Theil–Sen approximation.

The scatterplot in Figure 1 was fitted with the multi-segment Theil–Sen regression model. The number of segments, coefficient of regression lines, and the convergence points were calculated and adjusted according to the procedure described by Granato [50]. The two-line model, without discontinuity plotted on the scatterplot, was a good fit. One can notice that this relationship differs considerably for the CCR variable up to 7 and above. It can thus be well represented by two straight lines, i.e., by two-segment approximation. The Kendall tau-b for the first of approximated lines (CCR < 7) amounts to −0.1274, which confirms the negative trend. The low *p*-value, below 0.05, confirms that these results are statistically significant. The slope of the approximated line is −0.4851. Such a high value of the approximated slope indicates that the GDP is very sensitive to any changes of the CCR variable in the studied range. The relationship between GDP and CCR in the second of the identified ranges (CCR > 7) was statistically insignificant as the *p*-value was above 0.3. These results are endorsed by the Theil–Sen trend line, which was flat (the slope was 0.008), indicating that there was no relationships between CCR and GDP in the second range, i.e., for CCR above 7.

**Figure 2:**
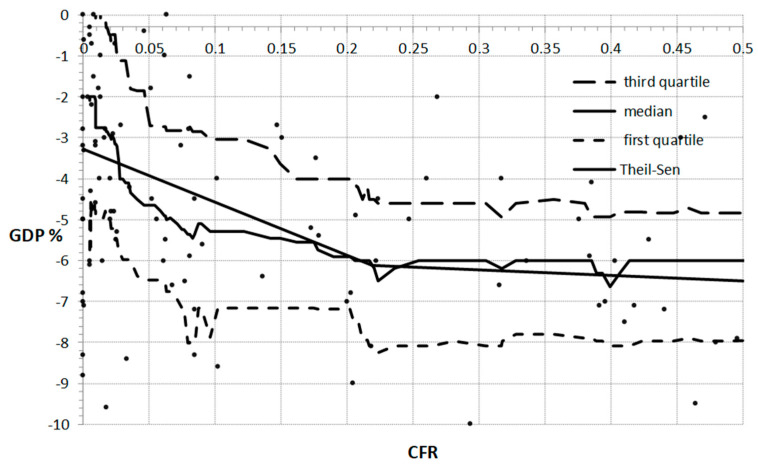
The relation between CCR and GDP with the moving median, first and third quartiles, and two-segment linear Theil–Sen approximation.

Figure 2 shows the relation between SARS-CoV-19 fatality rate (CFR) and GDP. As can be seen, the contemplated relationship can be divided into two different ranges and approximated with two straight trend lines. The first range comprised CFR observations below 0.2. Here, the Theil–Sen trend line (m = −12.9928) was sharp and fast declining. The relationship between CFR and GDP was negative. The Kendall tau-b coefficient was −0.1401. This confirmed that this relationship was negative and statistically significant (*p*-values below 0.05). The relation between CFR and GDP for GDP> 0.2 was well fitted by the Theil–Sen straight line with the slope of m = - 1.3475 and the intercept b = −5.8339. This result was endorsed by the Kendall tau-b, which was −0.1421. The results are statistically significant with *p* = 0.0456.

## 4. Discussion

As already discussed and confirmed in the literature, the global health crisis caused by coronavirus-2019 has negatively affected the world economy. This negative impact has already been discussed at the levels of both local economies [19,20,21] and globally [22,23,24]. Several researchers have provided the very first approximations of the global economic costs of the SARS-CoV-19 pandemic, which might total at around USD 2.7 trillion [54]. Other scholars note that the negative impact of SARS-CoV-19 on the economies has not only caused GDPs to decline but has also increased the importing and exporting costs. At the same time, it must be added that this negative impact varies greatly between respective economy sectors, with tourism and domestic services losing the most, and natural resources and agriculture being only negligibly affected. Reports also confirm considerable differences between individual countries [55]. König and Winkler provided the initial empirical evidence on the relationship between the SARS-CoV-19 pandemic and the economic crisis. Their research was based on data from the first three quarters of 2020 relating to 42 countries, using simple ordinary least squares and panel fixed effects regressions. They explained the link between the pandemic and the GDP by highlighting two major factors. The first results from restrictive measures imposed by the governments of different countries. Their results indicate that governmental restrictions lowered the GDP growth in the same quarter, with noticeable improvement in GDP dynamics in the following one. The second link between the pandemic and GDP is the health risk that leads to voluntary social distancing, which, again, lowers the GDP. They measured the health risk on the basis of fatality rates and provided initial empirical evidence suggesting that high fatality rates contribute significantly to the negative growth rates [56]. In comparison with the study by König and Winkler, our research was based on a considerably larger sample (176 versus 42 countries), related to a longer period (full year instead of three quarters), and employed a different econometric approach and databases. Our results are aligned to postulates of other scholars and empirically confirm the negative impact of the SARS-CoV-19 pandemic on the global economy. In our calculations, Kendall tau-B coefficients confirmed the negative correlation between SARS-CoV-19 fatality rates and changes in the GDP relative increase. The very low *p*-value (below 0.05) indicates that the obtained results are robust. We also identified a negative relationship between SARS-CoV-19 infections rate (CCR variable) and the decrease of gross domestic product. This relationship, however, was only significant for CCR values below 7, i.e., up to 7 infections per 1000 people in 2020. Therefore, the first of the formulated hypotheses, stating that *there is a negative correlation between the SARS-CoV-19 pandemic and GDP growth*, has been corroborated.

The onset of the SARS-CoV-19 pandemic and efforts aimed at limiting its further development were approached differently by decision makers in various countries. The tools most frequently employed against the pandemic included isolation, quarantine, and lockdown. In general, the more rapidly the pandemic developed in a country, the more severe restrictions were introduced by the policymakers [27,28]. SARS-CoV-19 tests have also been widely used in an effort to halt the pandemic development. Several researchers have claimed that the expansion of the pandemic can be limited by increasing the testing rate [57]. However, high costs and financial restraints caused the countries employ different testing strategies [34,58,59]. The third tool employed to reduce the negative impact of the SARS-CoV-19 pandemic on the economy entailed government programs supporting the companies and citizens during periods of isolation and lockdowns [29,30]. Finally, several scholars have also noted the development of interactions between different countries during the pandemic [31,32]. Assuming that all the aforementioned actions might affect the expansion of the pandemic, we formulated the second hypothesis, namely, that *the correlation between the pandemic and GDP fluctuations is not a linear one*. We estimated that the relationship between SARS-CoV-19 fatality rates and changes in the gross domestic product relative increase can be well fitted with two straight lines, with slopes of −12.9928 and −1.3475 and in the ranges below 0.2 deaths per 1000 (or up to 200 per 1 million) of the population and above the same, respectively. This shows that the GDP falls by 0.013 percentage points with each death per 1 million people. Once the threshold of 0.2 deaths per 1000 people is exceeded, however, the gross domestic product becomes less sensitive to new SARS-CoV-19 deaths and falls by only 0.0013 percentage points with each new death per 1 million people. The relationship between gross domestic product and SARS-CoV-19 morbidity is similar, with the threshold of 7 new infections per 1000 people. Since the slope of the approximated line was −0.4851, each new infected person per 1000 residents reduced the GDP by 0.4581. Once the threshold of seven new infections is exceeded, the contemplated relationship becomes statistically insignificant (very high *p*-value Kendall tau-B). Hence, the GDP change is considerably more sensitive to new SARS-CoV-19 deaths than to infections. The two distinctly identified ranges in the relationship between GDP and SARS-CoV-19 morbidity and fatality rates indicate that there is a threshold over which countries manage to adapt to the pandemic. Due to this observation, our second hypothesis must be accepted. The world economic crisis triggered by the SARS-CoV-19 pandemic is a complex and multifaceted issue and differs from other economic slowdowns in recent decades. This is because, additionally to the burden on the healthcare systems, the pandemic has directly resulted in the premature deaths of employees, large-scale workplace absenteeism, productivity reduction, negative supply shocks, manufacturing activity slow-down, global supply chain disruptions, and decimation of the tourism industry [60]. Given the complexity of this crisis, a fraction of researchers employed a high-level, comprehensive approach in their studies, while most of the others focused only on selected economy issues. Al-Thaqeb et al. claim economic policy uncertainty plays a key role in understanding the current crisis. They indicate that high uncertainty in terms of economic policy is associated with adverse effects on households, corporations, and governments, which tend to delay financial decisions due to the same reasons. This leads to lower consumption, lower loan issuance, fewer investments, and higher unemployment. They also note that the effects of political and regulatory uncertainty extend even to the commodity and crypto-currency markets. [61]. Song and Zhou also acknowledge the fact that uncertainty plays a key role in the current economic crisis. They draw attention to high periods prior to SARS-CoV-2, resulting primarily from Chinese economy problems and slow recent growth; synchronized global economy slowdown; de-globalization; and unfavorable macroeconomic settings, including deflation [62]. Stiifanić et al. studied the impact of the SARS-CoV-19 pandemic on crude oil prices and selected stock indexes: DJI, S&P 500, and NASDAQ. They employed new SARS-CoV-19 infections as the independent variable. In terms of crude oil prices, they identified uncertainty and supply shock increase in the global stocks of crude oil, which led to price slumping. They also demonstrated the existence of a link between stock market prices and the pandemic, indicating, however, that the movement of stock indexes does not reflect the real situation in the economy but is mainly based on expectations and monetary and fiscal incentives. [63]. Finally, other researchers studied the impact of the SARS-CoV-19 pandemic on selected industries, namely, travel, hospitality, sports, events, entertainment, education, and finance. Their approximations were negative for each of the studied industries [64]. In our study, we focused on the question of how the SARS-CoV-19 pandemic has affected the economies of 176 selected countries as a whole. Therefore, unlike other authors, we did not study specific industries or selected economy ratios. Instead, we considered the change in gross domestic product relative increase throughout the pandemic period in the studied countries, which we considered to provide a good proxy for the condition of economy as a whole. Our sample of countries and studied period were considerably larger than those reported in previous studies. As a result of the above and due to the robust statistical results, our findings provide global evidence on how the SARS-CoV-19 pandemic has affected the economies of respective countries. 

The presented study has several limitations. Firstly, the sample for this study comprised observations relating to 176 countries. Hence, not all countries could be considered in this study. Secondly, we calculated the change in the GDP for each country on the basis of the initial calculations of 2020 GDP provided by the International Monetary Fund as of 7 March 2021. These variables might require minor adjustments. The aforementioned two limitations might change the values of coefficients calculated in the study, but not our overall results. This is because our results were robust with low *p*-values (below 0.05). The final limitation of this study results from the analyzed period. Since we studied observations relating to only a single year, i.e., 2020, we are not in a position to conclude on the long-term impact of the SARS-CoV-19 pandemic on the economies of the analyzed countries.

In this study, we identified the economies of the countries with regards to the SARS-CoV-19 pandemic. We did not study the underlying reasons, which we consider to be rather multifaceted in nature. Hence, further research into adaptation mechanism in the context of the pandemic could prove interesting from the scientific point of view. In this paper, we analyzed and identified global trends related to the pandemic and GDP fluctuations. To this end, we ignored local differences, timing, and intensity of the pandemic. Although such an analysis was not required for our study, we consider it to be a good indication for further research. The final indication for the continuation of our research is to study the long-term impact of coronavirus-2019 on the global economy.

## 5. Conclusions

The global health and economy crisis caused by coronavirus-2019 is unprecedented, both in terms of its global dimension and the depth of its impact on the economy, society, political and legal conditions, and even the environment. The severity of the aforementioned phenomena and various related factors continues to increase, while the pandemic’s prolonged duration poses a very real risk of a global catastrophe. As a result, many researchers and state policymakers have shifted the domains of their professional interests towards the pandemic and its surrounding issues.

In this paper, we studied data relating to 176 countries in the pandemic period and in the previous year. The objective of this paper was to study the impact of SARS-CoV-19 pandemic on national economies. Given the fact that at the time of writing this article real data pertaining to GDP values worldwide have just been officially provided, our study is the first or one of the first to analyze the impact of SARS-CoV-19 on the economies of countries in the first year of the pandemic. We selected the SARS-CoV-19 morbidity and fatality rates as proxies for the pandemic’s development and GDP fluctuations in 2020 and 2019 as representative of the state of economy. Firstly, we identified the negative and statistically significant correlations between the SARS-CoV-19 morbidity and fatality rates and GDP changes. Next, we determined that the studied relationships were not linear, with the economies growing increasingly immune to the pandemic. The more severe the development of the pandemic, the more adaptive to its negative effects the economies eventually became. This finding is especially interesting as it shows that the global economy can quickly adjust to fast-changing severe and negative conditions. Hence, our results also demonstrate how the economies of the respective countries have been developing immunity to the negative effects of coronavirus-2019. This has been due to both governmental action and agility of companies.

Our results might have practical implications for policymakers. We identified and econometrically measured the direct connection between the spread of the SARS-CoV-19 pandemic, measured with new infections and fatality rates, and the change in gross domestic product relative increase. Therefore, our results empirically confirm the intuitive approach of many policymakers, who were the first to assume that the spread of the virus should be contained and next the economic issues should follow.

Finally, it should be noted that our paper shows the possibility of applying the multi-segment Theil–Sen model to disciplines such as healthcare and economics.

## Figures and Tables

**Table 1:** Description of the study sample by continent.

Continent	New Cases	Deaths	Population	Number of Countries
Africa	2,753,352	65,360	1,333,308,499	53
Asia	19,659,852	334,364	4,518,306,798	41
Europe	23,796,060	545,361	748,015,042	42
North America	23,013,002	512,367	575,845,728	20
Australia and Oceania	31,440	945	41,417,217	8
South America	13,194,159	362,651	430,457,607	12
Total	82,447,865	1,821,048	7,647,350,891	176

**Table 2:** Variables used in the study.

Acronym	Variable	Description
CCR	SARS-CoV-19 cases rate	New SARS-CoV-19 cases reported in a country per 1000 of the population of the country
CFR	SARS-CoV-19 fatality rate	New SARS-CoV-19 deaths reported in a country per 1000 of the population of the country
GDP	Gross domestic product	The difference of gross domestic product in 2020 and 2019, divided by gross domestic product in 2019, calculated for each country

**Table 3:** Descriptive statistics.

Variable	Mean	SD	Median	Min	Max	Skewness	Kurtosis
CCR	15.749	18.874	7.263	0.003	76.819	1.239	0.659
CFR	0.295	0.392	0.083	0	1.739	1.42	1.181
GDP	−5.694	7.181	−5.1	−66.7	26.2	−3.271	30.415

**Table 4:** Results of statistical tests using the Kendall tau-b of relations between CCR, CFR, and the change in GDP relative increase (GDP) with Theil–Sen slopes (m) and intercepts (b) of trend lines approximating these relations.

		Kendall			Theil–Sen
Variable	Range	Tau-B	*p*	m Slope	b Intercept
Gross Domestic Product (GDP)					
CCR	<7	−0.1274	0.0383	−0.4851	−2.7409
CCR	>7	−0.0339	0.3231	−0.008	−6.2276
Gross Domestic Product (GDP)					
CFR	<0.2	−0.1401	0.0161	−12.9928	−3.2868
CFR	>0.2	−0.1421	0.0456	−1.3475	−5.8339

## Data Availability

The data used in this study were from the open access databases “Our World in Data” and “International Monetary Fund” and can be found there. Further information on data and materials used are available from the corresponding author on reasonable request.
